# You See What You Smell: Preferential Processing of Chemosensory Satiety Cues and Its Impact on Body Shape Perception

**DOI:** 10.3390/brainsci11091152

**Published:** 2021-08-30

**Authors:** Bettina M. Pause, Annika S. Schäfer, Matthias Hoenen, Katrin T. Lübke, Ursula Stockhorst

**Affiliations:** 1 Department of Experimental Psychology, Heinrich-Heine-University Düsseldorf, D-40225 Düsseldorf, Germany; annika.schaefer@hhu.de (A.S.S.); katrin.luebke@hhu.de (K.T.L.); 2 FOM University of Applied Sciences, D-45141 Essen, Germany; matthias.hoenen@fom.de; 3 Institute of Psychology, Experimental Psychology II and Biological Psychology, University of Osnabrück, D-49074 Osnabrück, Germany; ursula.stockhorst@uos.de

**Keywords:** satiety, fasting, diet, metabolic state, BMI, body odors, chemosensory communication, olfaction, chemosensory cues, event-related potentials

## Abstract

The current study examines neural responses to satiety- and fasting-related volatiles and their effect on the processing of body shapes. Axillary sweat was sampled with cotton pads from 10 individuals after 12 h of fasting, and after having consumed a standard breakfast. Pure cotton pads served as the control. The chemosensory stimuli were presented to 20 participants (via a constant-flow olfactometer) exclusively, and additionally as context to images of overweight and underweight avatars. EEG was recorded (61 electrodes), and chemosensory (CSERPs; P1, N1, P2, P3) and visual event-related potentials (VERPs; N1, P2, P3a, P3b) were analyzed. The amplitudes of all positive CSERP components differed more strongly from cotton in response to chemosensory satiety cues as compared to fasting cues (P1: *p* = 0.023, P2: *p* = 0.083, P3: *p* = 0.031), paralleled by activity within the middle frontal and temporal gyrus. Overweight compared to underweight body shapes tended to elicit larger VERP P2 amplitudes (*p* = 0.068), and chemosensory satiety cues amplified the VERP amplitudes in response to any body shape (P2, P3a, P3b; all *p_s_* ≤ 0.017) as compared to the cotton control. The results indicate that chemosensory satiety cues transmit complex social information, overriding the processing of analogous visual input.

## 1. Introduction

Humans exchange various kinds of social information via chemosensory communication, related to the status of the genetic, neuronal, endocrine, and immune systems [1,2,3]. Hereby, social volatiles convey messages about age, gender, health, personality, basic drives, and emotions, fostering reproductive success and harm avoidance in humans.

An effect of metabolic disorders on body odors was already assumed in ancient Greek and Hindu medicine [4,5]. Depending on the deficient enzymes or transport systems, metabolic disorders such as phenylketonuria or diabetes affect the smell of urine, sweat, or breath [6]. However, in addition to metabolic disorders, metabolic activity also induces transient changes in body odor quality. While it is well known that fruits and vegetables like garlic or asparagus strongly affect the odor of breath, sweat, and urine [6], there is only minor knowledge regarding the fact that food consumption habits also affect the emotional valence of body odor. The body odor of men regularly consuming red meat is rated as being more unpleasant and more intense than body odor samples from men on a non-meat diet [7]. Similarly, the axillary odor of men consuming a diet rich in fruits and healthy vegetables, e.g., garlic, is associated with an increase in pleasantness [8,9]. Accordingly, studies in several animal species demonstrate that high-quality diets enriched with proteins or vitamins increase the attractiveness of male body odor for conspecific females (e.g., [10,11,12,13]). It has been assumed that well-fed males release chemosensory cues for the benefit of females in the selection of high-quality mates, as these cues represent access to healthy food sources.

In line with the findings regarding the effects of food quality on body odor, long-term food deprivation of males [14] or their mothers during gestation [15] decreases the attractiveness of their body odor, indicating undernourishment and susceptibility to disease. To our knowledge, only one study has investigated the effects of fasting on body odor in humans [16]. Here, axillary sweat samples were taken from women before and right after a 48 h fasting period, and 72 h after restoration of caloric intake. Unexpectedly, the pleasantness and attractiveness of body odors did not change during fasting as compared to the baseline but increased after the restoration period in comparison to the baseline by male raters. Thus, in deducing possible health effects from fasting periods, it seems to be necessary to separate effects of short-term fasting from effects of long-term mal- and undernutrition. Short-term fasting in animals and humans has consistently been described to improve physical and mental health, thereby reducing the mortality rate [17,18]. Accordingly, the effects on body odor quality may vary with the fasting duration.

The aim of the present study was to investigate whether short-term (12 h) fasting in comparison to food restoration by breakfast consumption affects body odor quality. This effect should be evident in a changed body odor quality perception and in the pattern of central nervous processing of body odors. It is to be expected that chemosensory satiety cues contain more information on the metabolic state than cues of short-term fasting. Therefore, chemical cues related to food consumption are considered to require more neuronal processing resources than chemosignals obtained after a brief fasting period. Second, by introducing the body odors as context cues, changes in the visual perception of human body shapes should be evident. Here, pictures of overweight and underweight avatars were presented in the context of satiety and fasting body odor. Several studies demonstrate that social chemosignals are able to change the information content of social visual signals in multimodal settings, suggesting a prominent role of chemosignals in social communication [19]. In line with the effects of anxiety and fear odor in easing the social visual perception of fear [20], reducing the perceptual acuity for cues of happiness [21], and increasing the perceptual relevance of neutral human actions [22], it is assumed that chemosensory satiety cues adjust the visual processing of underweight avatars and that chemosensory fasting cues adjust the visual processing of overweight avatars, each according to the chemosensory context.

## 2. Materials and Methods

### 2.1. Participants

In total, 22 individuals (13 women, 9 men) participated in the experiment. For the analyses of the chemosensory stimuli, data of 2 men had to be excluded because they did not comply with the breathing instructions (see Section 2.5, Data Reduction). The remaining 20 participants (13 women, 7 men) had a mean age of 23.0 years (SD = 2.9 years, range = 19–29 years), a mean body mass index (BMI) of 23.0 kg/m^2^ (SD = 2.6 kg/m^2^, range = 18.2–29.4 kg/m^2^), and rated their feeling of hunger prior to the EEG recording (0 = “not hungry at all” to 100 = “very hungry”) on average as M = 15.6 (SD = 19.5, range = 0–55).

Furthermore, for the analyses of the visual stimuli and the avatars’ BMI recollection task, the data of 2 women had to be excluded because of a failure to comply with the breathing instructions (see Section 2.5, Data Reduction). The remaining 20 participants (11 women, 9 men) had a mean age of 23.1 years (SD = 3.0 years, range = 19–29 years), a mean body mass index of 23.0 kg/m^2^ (SD = 2.6 kg/m^2^, range = 18.2–29.4 kg/m^2^), and rated their feeling of hunger on average as M = 16.2 (SD = 19.4, range = 0–55).

All participants were right-handed (assessed using, [23]), non-smokers, and of European descent (minimizing effects of culture, ethnos, and genetic background). In addition, no participant reported having had any previous nasal surgery or suffering from any neurological, psychiatric, endocrine, or immunological condition, or diseases related to the upper respiratory tract. None of the participants reported receiving acute or chronic medication (except for 9 females taking oral contraceptives), or using drugs. No participant was on a diet, fasted, or followed a vegetarian or vegan diet at the time of participation or within the week before.

Visual acuity was assessed using Landolt rings (EN ISO 8596) and was always better than 80% normal vision. A brief olfactory screening test revealed no suspicion of general hyposmia in any participant. The test required the participants to detect phenylethyl alcohol (99%, 1:200 (*v*/*v*) diluted in diethyl-phthalate (99%)—both substances from Fluka, Germany) present in 1 of 3 brown glass bottles in 2 consecutive trials, with the remaining 2 bottles containing the same volume of solvent. Phenylethyl alcohol smells like roses, and is regularly used as a standard in olfactory sensitivity testing.

All participants gave their written informed consent and were paid for their participation. The study was conducted in accordance with the Declaration of Helsinki and was approved by the ethical committee of the Faculty of Mathematics and Natural Sciences of the Heinrich-Heine-University Düsseldorf, Germany (LU01-2021-01). The sweat donation procedure was approved by the ethics committee of the University of Osnabrück, Germany (approved 37/5, 2012, extended 38/10, 2013).

### 2.2. Materials

#### 2.2.1. Chemosensory Stimuli

The study comprised 2 parts: the sampling of axillary sweat (conducted at the University of Osnabrück), and the main study with preparation of the chemosensory stimulus material and conducting the main protocol (conducted at the University of Düsseldorf).

Axillary sweat was sampled from 6 female and 6 male donors. However, the samples of 2 donors had to be excluded because the respective donors reported having used fragrant cosmetics. The remaining 10 donors (5 women, 5 men) had a mean age of 23.8 years (SD = 1.7 years, range = 21–26 years). Female donors had a mean BMI of 22.5 kg/m^2^ (SD = 1.4 kg/m^2^, range = 20.4–24.0 kg/m^2^), and male donors had a mean BMI of 22.1 kg/m^2^ (SD = 1.6 kg/m^2^, range = 19.6–23.9 kg/m^2^). These donors’ sweat samples were additionally checked for any conspicuous smells and appeared not to be contaminated with fragrances.

All donors reported being non-smokers, not receiving acute or chronic medication, and not using drugs. They further reported not suffering from any chronic or acute mental or physical conditions. No donor was on a diet, followed a vegetarian/vegan diet, or had any food aversion. Female donors reported not being pregnant and not being in a lactation period. All donors gave written informed consent and were paid for their participation. During their laboratory task, the fasting sweat cotton pads were collected. The participants consumed a standardized breakfast and took part in an assessment of their olfactory and gustatory performance; and blood glucose samples were collected as described below.

No later than 2 days before the donation, all donors trimmed or shaved their armpits. The night before the donation, the donors had to wash their armpits with an unperfumed medical soap (Eubos^®^, Dr. Hobein GmbH, Meckenheim, Germany). Thereafter, they were only allowed to wash their armpits exclusively with water and had to refrain from using deodorant or other products such as perfumes or shaving foam. Within the 24 h prior to donation, the donors were instructed to refrain from eating garlic, onions, asparagus, or spicy food, and from drinking alcohol. Exactly 12 h before donation, the donors reported having finished a regular dinner which had lasted 30 min at most. Afterwards, they only consumed water or unsweetened fruit tea for 12 h (short-term fasting period). On the day of donation, 2 h before the start of the sessions the donors applied cotton pads for the fasting sweat condition at home, following precise instructions (fasting sweat). They had been instructed to avoid any arousal-inducing physical activity during these 2 h, and thus the fasting and the satiety sweat condition should not have differed due to physical activity. After the donors arrived at the laboratory, the cotton pads were removed (donation duration: M = 126 min, SD = 2 min). The participants were interviewed about their compliance regarding the behavioral restrictions of personal hygiene, physical activity, and diet. Except for the 2 donors reporting the use of fragrant cosmetics (see above), no donor had to be excluded. All donors then ate a standardized carbohydrate-rich breakfast (total calories: 608) within 10 min (Table 1).

The breakfast’s carbohydrate-rich composition was chosen to guarantee a fast and reliable increase in glucose and insulin [24]. Exactly 8 min after the end of breakfast, the cotton pads for sweat for the satiety condition were applied and worn for an average of 120 min (SD = 1 min).

**Figure 1:**
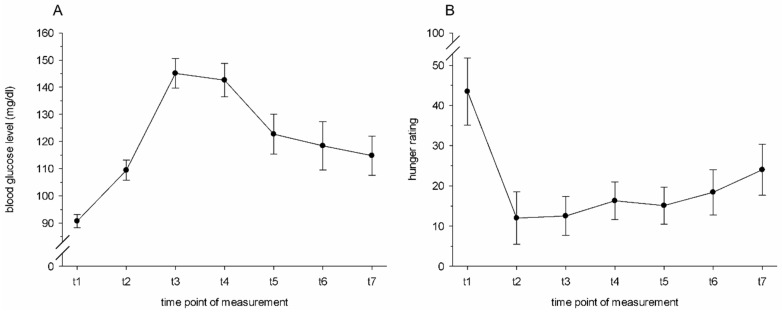
Sweat donors’ mean (±SEM) blood glucose levels (**A**) and hunger ratings (**B**).

At a total of 7 timepoints, the donors’ hunger level was assessed, and a capillary blood sample was taken from the fingertips of the non-dominant hand using disposable lancets (ACCU-CHEK^®^ Safe-T-Pro Plus, Roche Diagnostics, Mannheim, Germany). A glucometer device from Roche (ACCU-CHEK^®^ Aviva, Roche Diagnostics, Mannheim, Germany) and the corresponding disposable test strips (also ACCU-CHEK^®^ Aviva) served to measure capillary blood glucose levels at 5 min prior to the beginning of the breakfast (t1), and then again at 15 (t2), 30 (t3), 45 (t4), 68 (t5), 86 (t6), and 105 (t7) minutes after the donors had started their breakfasts. The hunger level was rated via a visual analogue scale, ranging from 0 (“not hungry at all”) to 100 (“extremely hungry”). At each timepoint after the standardized breakfast (t2–t7), the donors had a higher blood glucose level compared to prior to the standardized breakfast (t1, all *p_s_* ≤ 0.006, 1-sided; Figure 1A). Accordingly, the donors reported being hungrier before the breakfast (t1) compared to each timepoint after the breakfast (t2–t7, all *p_s_* ≤ 0.031, 1-sided, Figure 1B).

The cotton pads were stored at −20 °C until being cut and pooled across donors with respect to the given chemosensory condition (satiety sweat, fasting sweat). Additionally, samples of pure cotton were treated the same way (cotton). The homogenized samples were divided into portions of 0.4 g, packed in aluminum foil, and stored at −20 °C.

#### 2.2.2. Visual Stimuli

**Figure A1:**
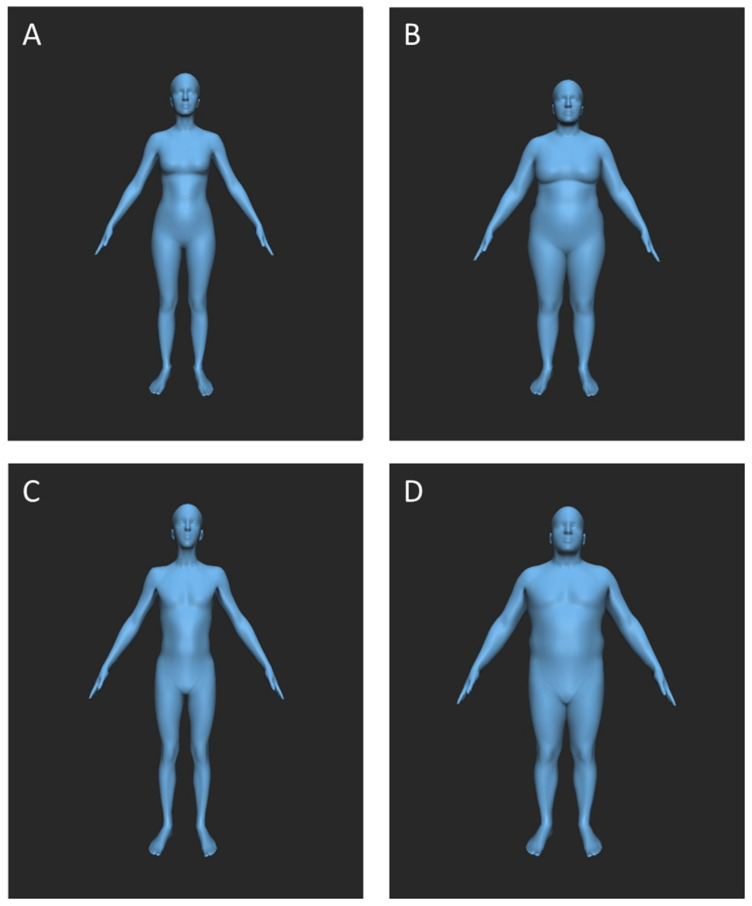
Examples of underweight (**A**,**C**; BMI = 10.5 kg/m^2^) and overweight (**B**,**D**; BMI = 39.5 kg/m^2^) female (**A**,**B**) and male (**C**,**D**) avatars [25].

The visual material used in this study consisted of images showing human avatars with different body shapes. For EEG recordings, 20 overweight (10 male, 10 female, BMI of 34.9–39.5 kg/m^2^) and 20 underweight (10 male, 10 female, BMI of 10.3–15.2 kg/m^2^) avatars were created using the BMI Visualizer [25], with BMI increments of 0.25 kg/m^2^ between the images. The BMI Visualizer “uses a statistical model of human body shapes created from thousands of detailed laser range scans of human bodies”, taking gender into account (http://bmijs.is.tuebingen.mpg.de/en/main/about, accessed on 23 August 2021). The avatars’ body height was selected based on the average German height [26]. The avatars were shown in frontal view with their arms out to the side and their legs hip-width apart (see Appendix A, Figure A1). For the estimation of the sweat donors’ BMI and the recollection of the avatars’ BMI, another 101 male avatars were used in order to visualize smoothly changing, adjustable BMIs ranging from 12.5 to 37.5 kg/m^2^ (see Section 2.2.4, Odor Discrimination, Odor Ratings, Estimation of the Sweat Donors’ BMI, Recollection of the Avatars’ BMI). These avatars were presented in a 45° side view to prevent the participants from recalling a given body shape from sensory memory during the recollection of the avatars’ BMI task.

#### 2.2.3. Presentation of the Chemosensory and Visual Stimuli

The chemosensory stimuli were presented during the inhalation phase of the breathing cycle [27], using a constant air-flow (100 mL/s) 6-channel olfactometer (OM6b, Burghart GmbH, Wedel, Germany, built in accordance with [28]). Both nostrils were stimulated simultaneously, and both air streams were controlled by separate mass-flow meters. In the olfactometer, the glass tubes containing the stimuli (0.4 g of each stimulus per nostril) were stored in a warm-water chamber, and the stimuli were delivered to the participants through a Teflon tube. The latency between the computer-controlled activation of the switching valve and the airflow reaching the mucosa was about 50 ms. The temperature of the air flow at the exit of the olfactometer was adjusted to body temperature (37.8 °C), and the relative humidity was set above 80%. Pink noise (dB(A) = 75) was presented binaurally over earplugs (ER3-14A, Etymotic Research, Elk Grove Village, IL, USA) to prevent the participants from hearing the switching valves of the olfactometer.

Visual stimuli were presented on a TFT monitor at a distance of 80 cm from the participants’ eyes. The experiment was controlled by Presentation 16 (Neurobehavioral Systems Inc., Berkeley, CA, USA).

#### 2.2.4. Odor Discrimination, Odor Ratings, Estimation of the Sweat Donors’ BMI, Recollection of the Avatars’ BMI

Odor discrimination, odor ratings, and the estimation of the sweat donors’ BMI were assessed prior to the EEG recordings, and the chemosensory stimuli were administered via the olfactometer (stimulus duration = 5000 ms). Avatar BMI recollections were included within the EEG procedure.

In order to infer whether the sweat samples presented were above or below the level of conscious perception, it was determined to what degree the participants were able to consciously discriminate satiety sweat, fasting sweat, and cotton, respectively. The participants were presented with 3 chemosensory stimuli per trial, 2 of which were identical. The task was to identify the stimulus that was different from the 2 identical stimuli. In 3 consecutive trials each, either satiety sweat had to be discriminated from 2 presentations of cotton, fasting sweat had to be discriminated from 2 presentations of cotton, and in addition, fasting sweat had to be discriminated from the 2 presentations of satiety sweat. The order of presentations was randomized in each trial. Participants who failed once to discriminate the relevant sweat sample from cotton were considered not to be able to smell the respective chemosensory stimulus.

The participants further judged the odor quality of the chemosensory samples with regard to intensity, pleasantness, unpleasantness, and familiarity, using pictographic computerized 9-level Likert-type scales (1 = “not perceivable”, “not pleasant”, “not unpleasant”, “not familiar“, respectively; 9 = “very intense”, “very pleasant”, “very unpleasant”, “very familiar“, respectively, cf. [29]).

In order to infer whether the chemosensory stimuli would affect the conscious evaluation of body shapes, the participants were presented with each chemosensory stimulus separately, and then asked to shape an avatar whose BMI would match the BMI they suspected the respective sweat donor to have (estimation of the sweat donors’ BMI). By moving the cursor along a visual analogue scale, they could adjust the BMI of an avatar presented directly above the scale. The same scale was used for the recollection of the avatars’ BMI. During each trial of the EEG recording, the participants were asked to match the shape of an avatar to the avatar presented previously.

### 2.3. Procedure

All participants were tested individually. Prior to the EEG recordings, participants rated their feeling of hunger, and completed the odor discrimination task, the estimation of the sweat donors’ BMI, and then the odor quality judgements for the 3 chemosensory stimuli (satiety sweat, fasting sweat, cotton). Afterwards, the electrodes were adjusted, and the participants were instructed to relax and to blink and move as little as possible to minimize artifacts. Participants completed practice trials to train their breathing rhythm. They were instructed to inhale exactly from the end of the countdown until the end of the avatar’s presentation (3750–4000 ms). An ongoing EEG was recorded during 120 trials of stimulus presentation with 40 presentations of each chemosensory stimulus (satiety sweat, fasting sweat, cotton). During these trials, each of the 20 overweight and 20 underweight avatars was presented once in the context of each chemosensory stimulus. The stimuli were presented in randomized order, with the restriction of a maximum of 4 consecutive presentations of either under- or overweight avatars.

**Figure 2:**
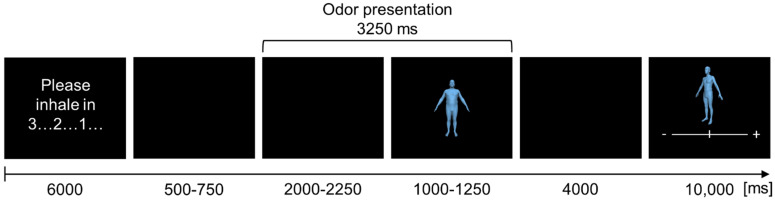
Schematic overview of the time sequence of one trial during an EEG recording.

At the beginning of each trial, the instruction “please inhale in…” appeared on the screen for 3000 ms, followed by a visual countdown from 3 to 1 (3000 ms; for an overview of a trial see Figure 2).

Afterwards, a black screen was presented for 2500–3000 ms (randomized). At 500–750 ms after the onset of the black screen, the switching valve was activated, and the chemosensory stimuli were presented for a total duration of 3250 ms. During the first 2000–2250 ms of this interval, only the chemosensory stimulus itself was presented (along with the black screen, to allow for the analyses of the chemosensory event-related potential (CSERP)). Then the presentation of the chemosensory stimulus continued alongside the presentation of the avatar for 1000–1250 ms (randomized). Afterwards, again a black screen was presented (4000 ms), followed by the presentation of the avatars’ BMI recollection task for 10,000 ms. Each trial had a mean duration of 23,875 ms (range = 23,750–24,000 ms). EEG recordings were subdivided into 4 blocks, separated by 3 individually adjusted resting periods. In total, the EEG recording lasted 57 min (SD = 5 min, range = 46–65 min).

### 2.4. Data Recording

The ongoing EEG was recorded using the Brain Vision Recorder 1.2 (Brain Products, Gilching, Germany) with 61 scalp locations with Ag/AgCl sintered ring electrodes. Electrodes were placed according to the extended 10/20 system using an electrode cap (EasyCap GmbH, Herrsching, Deutschland). An additional electrode was placed 1.5 cm below the right eye, outside the vertical pupil axis, to record the vertical eye movements. Fp2 was used to record the horizontal eye movements. The ground electrode was placed at position FT10. The impedance of the electrodes was always below 11 kΩ. Data were sampled at 500 Hz with an averaged reference, and low-pass filtered online at 140 Hz (Quick-Amp-72 amplifier and BrainVision Recorder software, version 1.2, Brain Products, Munich, Germany). Participants’ breathing cycles were assessed with 2 respiration belts (XactTrace Belt, Brain Products, Gilching, Germany), adjusted to the participants’ abdomen and thorax.

### 2.5. Data Reduction

#### 2.5.1. Chemosensory Event-Related Potentials

Offline, EEG signals were re-referenced to linked ear lobes, low-pass filtered with 40 Hz (48 dB/octave) and, to account for the slow build of the CSERP, high-pass filtered with 0.075 Hz (time constant: 2.1221 s, 48 dB/octave). Additionally, a 50 Hz notch filter was applied. The data recorded by the respiration belts were filtered with a 1 Hz low-pass filter (48 dB/oct). Data were then corrected for eye movements [30], and baseline-corrected (−500–0 ms before activation of the switching valve). Afterwards, trials during which participants failed to adhere to the breathing instructions were removed manually (valid trials had to include an inhalation beginning at least −200 ms prior to the activation of the switching valve and then lasting for at least 1000 ms). Two participants had to be excluded from analysis as fewer than 10 trials per chemosensory stimulus condition remained. In addition, trials contaminated by any further artifacts (amplitudes exceeding ±70 μV; maximum voltage difference in any 200 ms interval exceeding 50 μV) were eliminated from analysis. With respect to the relatively small amplitudes of the CSERP, the EEG signal was then low-pass filtered with 7 Hz in order to facilitate the peak detection (48 dB/octave; [31]). In relation to the baseline period (−500–0 ms before activation of the switching valve), 4 separate peaks were differentiated within predefined latency windows in electrodes Fz, Cz, and Pz (P1: 200–500 ms, N1: 300–600 ms, P2: 550–750 ms, P3: 750–1000 ms), and amplitudes and latencies of each peak were extracted. Since the current study is the first to analyze CSERP in response to fasting and satiety chemosignals, the time frames for the peak detection were based on [29], but were adjusted slightly after visual inspection of the aggregated grand average CSERP. Data of the remaining electrodes were retained for low-resolution electromagnetic tomography analysis (LORETA).

#### 2.5.2. Visual Event-Related Potentials

Data reduction in Fz, Cz, and Pz for calculating visual event-related potentials followed the same protocol as described. To account for the analysis of visual as opposed to chemosensory event-related potentials, adjustments were made to the filter range, the time frame of the inhalation validation, and the criteria during artifact rejection as follows: The low-pass filter was set at 30 Hz (48 dB/octave), and the high-pass filter at 0.25 Hz (time constant: 0.6366 s, 48 dB/octave). Adjusting the time frame of the inhalation validation to the presentation of the avatars, trials during which participants did not inhale beginning at least −500 ms prior to and lasting at least until 250 ms into picture presentation were removed manually. A total of 2 participants (not the same individuals as for the CSERPs) had to be excluded from analysis as fewer than 10 trials per condition remained. With respect to the relatively large amplitudes of the visual ERP, trials with amplitudes exceeding ±100 µV were further rejected as artifacts. In relation to the baseline period (−200–0 ms before stimulus onset), 4 separate peaks were differentiated within predefined latency windows based on the aggregated grand average (e.g., see [32,33]; N1: 90–170 ms, P2: 160–240 ms, P3a: 200–280 ms, P3b: 280–380 ms), and the amplitudes and latencies of each peak were extracted. LORETA was performed across all 61 electrodes.

### 2.6. Data Analysis

#### 2.6.1. Chemosensory Stimuli

Analysis of the CSERPs was based on difference values between satiety sweat and cotton (satiety sweat minus cotton), and short-term fasting sweat and cotton (fasting sweat minus cotton), using the responses to cotton as individual baselines. Difference amplitudes and latencies of the CSERP components (P1, N1, P2, P3) were analyzed using a 2 × 3 repeated-measures ANOVA with the within-subject factors Chemosensory Stimulus (satiety sweat minus cotton, fasting sweat minus cotton) and Electrode Position (Fz, Cz, Pz). Significant interactions were followed up by nested effects analysis [34] and, in the case of significant nested effects, simple comparisons (paired *t*-tests).

The analyses of the discrimination rates, the estimation of the sweat donors’ BMI, and odor ratings were based on *n* = 19, as data regarding 1 participant were not available due to technical issues. A 1-tailed binomial test was used to determine whether the proportion of individuals able to detect each chemosensory stimulus (satiety sweat, fasting sweat) was above the level of chance. In addition, McNemar’s test was calculated to determine differences between the discrimination rates of the chemosensory stimuli. The analyses of the estimation of the sweat donors’ BMI, and odor ratings (intensity, pleasantness, unpleasantness, familiarity) were also based on difference values between satiety sweat and cotton, and fasting sweat and cotton. The difference values were compared via *t*-tests.

In all analyses, the alpha level was set to α = 0.05. However, effects trending towards significance (*p* ≤ 0.10) are also mentioned. These effects are cautiously interpreted only in case that they support related significant effects. Huynh–Feldt corrected degrees of freedom were calculated, and corrected *p*-values are reported. For significant effects, η^2^_p_ as well as the achieved power were calculated. Statistical analyses were carried out using IBM SPSS Statistics (27.0; IBM Corp, Armonk, NY, USA).

LORETA was used in order to localize the source of brain activity [35]. The source space comprises 2394 voxels at a 7 mm spatial resolution, covering the cortical grey matter and the hippocampus [36], defined via a reference brain from the Brain Imaging Center at the Montreal Neurological Institute (MNI; [37]). LORETA uses a 3-shell spherical head model co-registered to the Talairach anatomical brain atlas [38]. Because LORETAs were created across all 61 electrodes, 3 additional participants had to be excluded due to eye movement artifacts (*n* = 17).

#### 2.6.2. Visual Stimuli

In contrast to the analyses of the responses to the chemosensory stimuli, cotton was retained as an original factor level in the analyses of the responses to the visual stimuli, allowing for interpreting responses to the avatars without any social chemosensory context. Based on a 2 × 3 × 3 ANOVA with the within-subject factors of Body Shape (overweight avatars, underweight avatars), Chemosensory Stimulus (satiety sweat, fasting sweat, cotton), and Electrode Position (Fz, Cz, Pz), it became evident that the N1 amplitude was dominant at Fz and Cz, and the 3 positive components P2, P3a, and P3b were each dominant at Cz and Pz (see Appendix A, Table A1). The factor of Electrode Position thus only includes the respective two dominant electrodes of the components. For visual event-related potentials, amplitudes and latencies of the components (N1, P2, P3a, P3b) were analyzed using a 2 × 3 × 2 repeated-measures ANOVA with the within-subject factors of Body Shape (overweight avatars, underweight avatars), Chemosensory Stimulus (satiety sweat, fasting sweat, cotton) and Electrode Position (N1: Fz, Cz; P2/P3a/P3b: Cz, Pz), followed up by nested effects analysis [34] and simple comparisons (paired *t*-tests). Main effects of the factor Electrode Position are not addressed explicitly in the Results section.

The recollection of the avatars’ BMI was analyzed using a 2 × 3 repeated measures ANOVA with the within-subject factors of Shape (overweight avatars, underweight avatars) and Chemosensory Stimulus (satiety sweat, fasting sweat, cotton). The analysis of the avatars’ BMI recollection task was based on *n* = 19, as data of 1 participant were not available due to technical issues.

All analyses were carried out with IBM SPSS Statistics using the same parameters as in the analyses of the chemosensory stimuli.

## 3. Results

### 3.1. Chemosensory Stimuli

#### 3.1.1. Stimulus Discrimination

In total, 3 out of 19 (15.80%) participants were able to discriminate satiety sweat from fasting sweat. Satiety sweat was detected by 5 out of 19 (26.30%) participants and fasting sweat by 2 out of 19 (10.50%). Participants’ discrimination performance did not exceed the level of chance for any chemosensory pairwise comparison (satiety sweat vs. fasting sweat: *p* = 0.998; satiety sweat vs. cotton: *p* = 0.968; fasting sweat vs. cotton: *p* > 0.999). Further, the discrimination rates did not differ between any two stimulus combinations (all *p_s_* > 0.375).

#### 3.1.2. Odor Ratings

Across all chemosensory stimuli (satiety sweat, fasting sweat, cotton), the participants judged the odor intensity as slightly below medium (M = 4.19, SD = 1.34), similar to odor pleasantness (M = 4.09, SD = 1.30), unpleasantness (M = 3.19, SD = 1.51), and familiarity (M = 3.53, SD = 1.44). There was no difference in the perceived intensity, pleasantness, unpleasantness, and familiarity between satiety sweat in reference to cotton and fasting sweat in reference to cotton (all *p_s_* > 0.299; for the descriptive difference values see Table 2).

#### 3.1.3. Estimation of the Sweat Donors’ BMI

On average, the participants estimated cotton “donors” to have a BMI of 26.3 kg/m^2^ (SD = 4.9 kg/m^2^, range = 17–34 kg/m^2^), satiety sweat donors to have a BMI of 23.1 kg/m^2^ (SD = 4.9 kg/m^2^, range = 17–34 kg/m^2^), and fasting sweat donors to have a BMI of 22.1 kg/m^2^ (SD = 4.9 kg/m^2^, range = 13–29 kg/m^2^). There was no difference in the estimated BMI between satiety sweat in reference to cotton and fasting sweat in reference to cotton (*p* = 0.622; for the descriptive differences values see Table 2).

#### 3.1.4. Chemosensory Event-Related Potentials

##### Amplitudes

**Figure 3:**
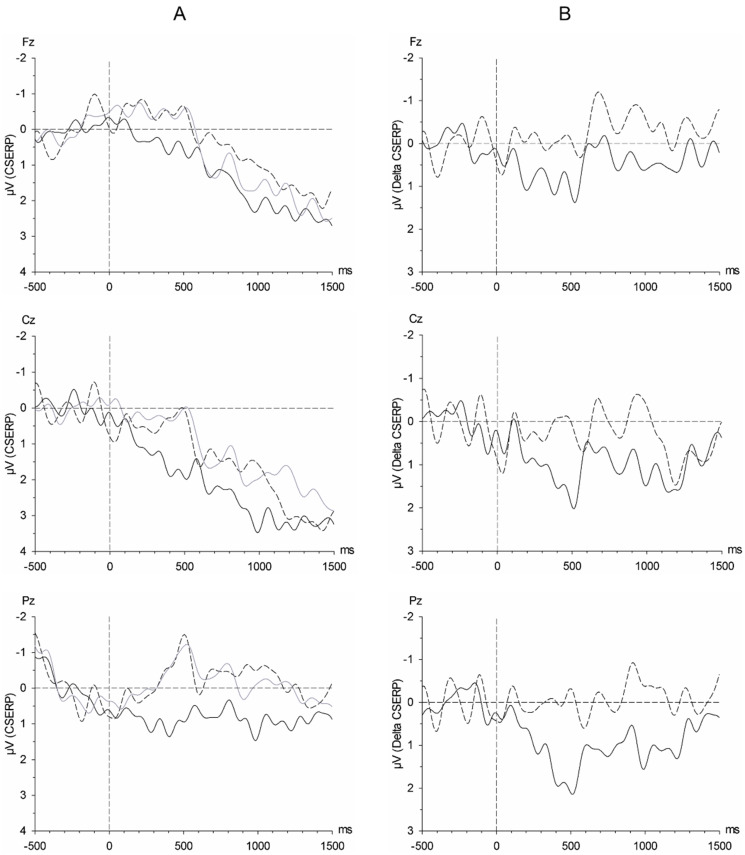
(**A**) Grand average of the chemosensory event-related potentials (CSERPs) in response to satiety sweat (solid black line), fasting sweat (dashed black line), and cotton (solid grey line). (**B**) Grand average of the difference chemosensory event-related potentials (Delta CSERPs) in response to satiety sweat in reference to cotton (solid line), and fasting sweat in reference to cotton (dashed line). Timepoint 0 refers to the activation of the switching valves.

Throughout all detected components, the differences between the positive amplitudes (P1, P2, P3) in response to satiety sweat vs. cotton were larger than the differences between the amplitudes in response to fasting sweat vs. cotton (expressed as Delta CSERPs, Figure 3). In detail, the participants showed a larger difference P1 amplitude in response to satiety sweat in reference to cotton as compared to fasting sweat in reference to cotton (Chemosensory Stimulus: F(1, 19) = 6.14, *p* = 0.023, η^2^_p_ = 0.244, power = 0.650; Figure 3). The N1 amplitude, on the other hand, was smaller (less negative) in response to satiety sweat in reference to cotton, while it was larger in response to fasting sweat in reference to cotton (Chemosensory Stimulus: F(1, 19) = 4.42, *p* = 0.049, η^2^_p_ = 0.189, power = 0.513, Figure 3). The difference P2 amplitude shows a pattern similar to the P1: It tended to be larger in response to satiety sweat in reference to cotton than in response to fasting sweat in reference to cotton (Chemosensory Stimulus: F(1, 19) = 3.35, *p* = 0.083, η^2^_p_ = 0.150, power = 0.412, Figure 3). This effect also was evident in the P3, appearing with larger difference amplitudes in response to satiety sweat as compared to fasting sweat (Chemosensory Stimulus: F(1, 19) = 5.41, *p* = 0.031, η^2^_p_ = 0.222, power = 0.596, Figure 3). The electrode position did not affect the difference amplitudes within any component (all *p_s_* > 0.244).

##### Latencies

Neither the Chemosensory Stimulus (all *p_s_* ≥ 0.286) nor the Electrode Position (all *p_s_* ≥ 0.101) affected the difference latencies within any component.

##### Low-Resolution Electromagnetic Tomography Analysis

**Figure 4:**
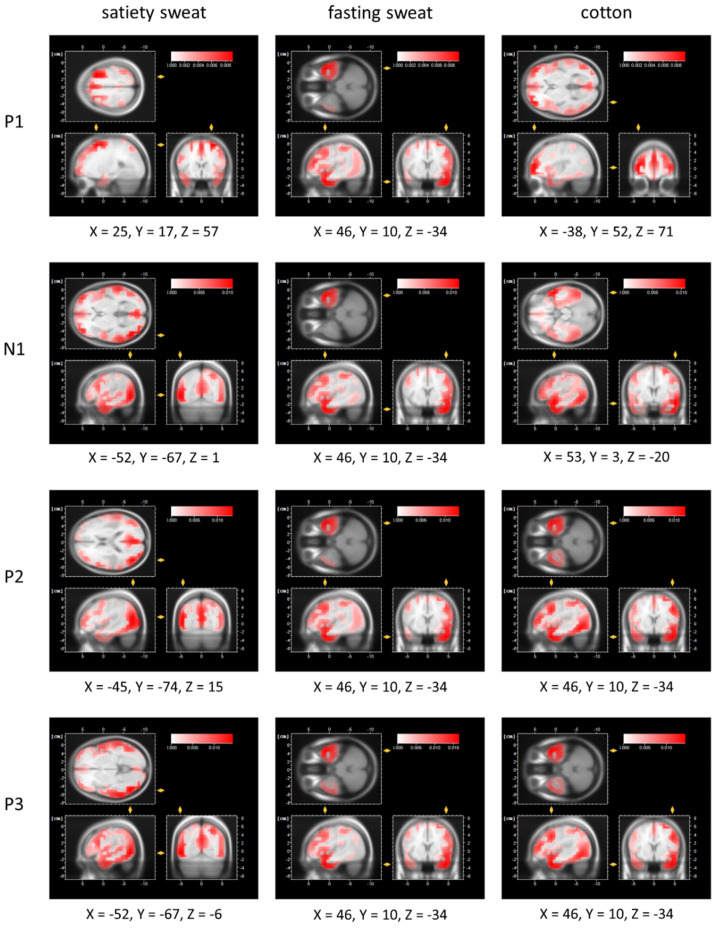
Low-resolution electromagnetic tomography analysis (LORETA) maps depicting the location of the maximum current density (in μA/mm^2^) upon the presentation of satiety sweat, fasting sweat, and cotton at the time of the individual mean P1 (satiety sweat = 358 ms, fasting sweat = 328 ms, cotton = 352 ms), N1 (satiety sweat = 480 ms, fasting sweat = 444 ms, cotton = 460 ms), P2 (satiety sweat = 654 ms, fasting sweat = 654 ms, cotton = 684 ms), and P3 latencies (satiety sweat = 892 ms, fasting sweat = 874 ms, cotton = 896 ms).

LORETA revealed that, throughout all stages of stimulus processing (P1, N1, P2, P3), exposure to satiety sweat elicited the most prominent neural activation within cortical areas differing from those activated by fasting sweat and cotton. In detail, exposure to fasting sweat and cotton was generally accompanied by activation within Brodmann area (BA) 21 localized in the middle temporal gyrus (see Figure 4). In contrast, in response to satiety sweat, the largest neural activity during early processing was evident in BA6 (right middle frontal gyrus, P1 time frame), BA37 (left middle temporal gyrus, N1 time frame), and BA 39 (left angular gyrus, P2 time frame, Figure 4). During the late processing stages, the most pronounced neural responses to satiety sweat occurred within BA19 (left inferior temporal gyrus, P3 time frame; Figure 4).

### 3.2. Visual Stimuli

#### 3.2.1. Recollection of the Avatars’ BMI

The participants remembered the overweight avatars (M = 34.1 kg/m^2^, SD = 1.7 kg/m^2^) as being more corpulent than underweight avatars (M = 20.0 kg/m^2^, SD = 1.7 kg/m^2^, Body Shape: F(1, 19) = 1064.12, *p* < 0.001, η^2^_p_ = 0.982, power > 0.999), validating the avatars’ perceptual properties. The recollected BMI of the avatars was unaffected by the chemosensory stimuli (all *p_s_* > 0.224).

#### 3.2.2. Visual Event-Related Potentials

##### Amplitudes

For a comprehensive overview of the ANOVA results see Table 3.

**Figure 5:**
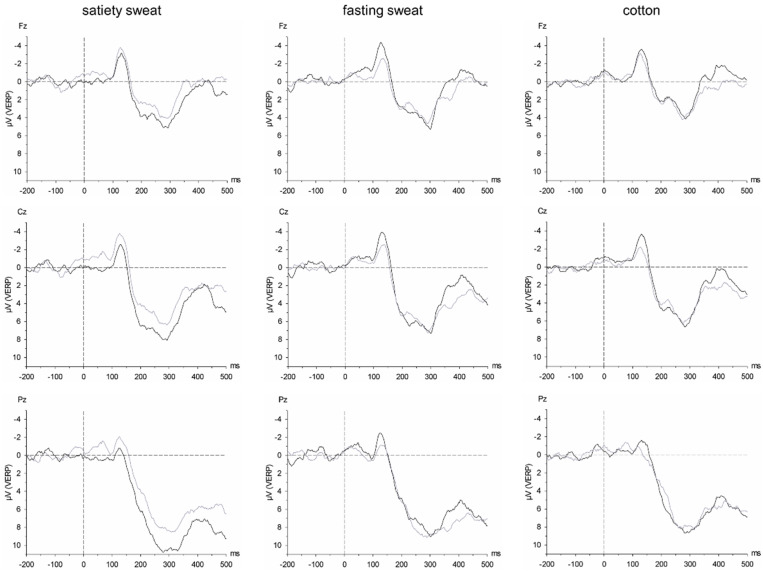
Grand average of the visual event-related potentials (VERPs) in response to overweight (black line) and underweight avatars (grey line) in the context of satiety sweat (**left** column), fasting sweat (**middle** column), and cotton (**right** column) at Fz (upper row), Cz (middle row), and Pz (lower row). Timepoint 0 refers to the onset of the avatars’ presentation.

When exposed to only cotton as chemosensory context, the participants tended to show larger central N1 amplitudes in response to overweight as compared to underweight avatars (Body Shape × Chemosensory Stimulus × Electrode Position: F(2, 38) = 4.06, *p* = 0.025, η^2^_p_ = 0.176, power = 0.687; nested effects: Body Shape × Chemosensory Stimulus within Cz: F(2, 38) = 3.50, *p* = 0.040, η^2^_p_ = 0.155, power = 0.618; Body Shape within cotton within Cz: F(1, 19) = 3.11, *p* = 0.094, η^2^_p_ = 0.141, power = 0.388; Figure 5).

However, when sweat samples were introduced as the chemosensory context, the N1 amplitude was generally the largest when participants were presented with any incongruent combination of social stimuli (overweight avatars paired with fasting sweat or underweight avatars paired with satiety sweat). In the context of satiety sweat, underweight avatars elicited larger central N1 amplitudes than overweight avatars (nested effects: Body Shape within satiety sweat within Cz: F(1, 19) = 5.35, *p* = 0.032, η^2^_p_ = 0.220, power = 0.591, Figure 5). In contrast, in the context of fasting sweat, frontal N1 amplitudes were larger in response to overweight compared to underweight avatars (nested effects: Body Shape × Chemosensory Stimulus within Fz: F(2, 38) = 4.00, *p* = 0.027, η^2^_p_ = 0.174, power = 0.680; Body Shape within fasting sweat within Fz: F(1, 19) = 9.80, *p* = 0.006, η^2^_p_ = 0.340, power = 0.842; Figure 5). Consistently, participants showed larger central N1 amplitudes in response to overweight avatars presented in the context of fasting sweat (and cotton) as in the context of satiety sweat (based on the same interaction Body Shape × Chemosensory Stimulus × Electrode Position; nested effects: Chemosensory Stimulus within overweight avatars within Cz: F(2, 38) = 4.31, *p* = 0.022, η^2^_p_ = 0.185, power = 0.715; satiety sweat vs. fasting sweat: t (19) = 2.29, *p* = 0.034, d = 0.512, power = 0.585; satiety sweat vs. cotton: t (19) = 2.44, *p* = 0.025, d = 0.545, power = 0.638).

**Figure 6:**
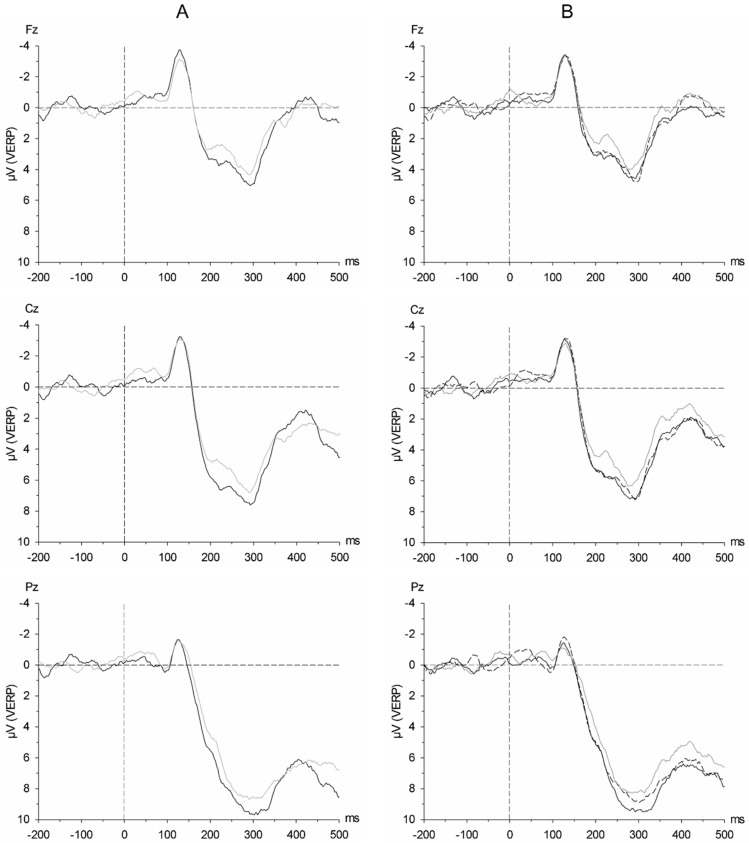
(**A**) Grand average of the visual event-related potentials (VERPs) in response to overweight (black line) and underweight avatars (grey line) across all chemosensory context stimuli. (**B**) Grand average of the visual event-related potentials (VERPs) in response to all avatar shapes in the context of satiety sweat (solid black line), fasting sweat (dashed black line), and cotton (solid grey line). Timepoint 0 refers to the onset of the avatars’ presentation.

In general, the P2 amplitude tended to be more pronounced in response to overweight compared to underweight avatars, irrespective of the chemosensory context (Body Shape: F(1, 19) = 3.75, *p* = 0.068, η^2^_p_ = 0.165, power = 0.451; Figure 6A). Similarly, the P2 amplitude also tended to be more pronounced in response to any of the avatars presented in the context of satiety sweat as compared to cotton, irrespective of the avatars’ shape (Chemosensory Stimulus: F(2, 38) = 2.50, *p* = 0.100, η^2^_p_ = 0.116, power = 0.471; satiety sweat vs. cotton: t (19) = 2.62, *p* = 0.017, d = 0.585, power = 0.699; Figure 6B).

The pattern of pronounced responses to any of the avatars presented within the context of satiety sweat, irrespective of their body shape, remained evident throughout later processing stages. Both the P3a and the P3b component had larger amplitudes (at Pz within the P3b) in response to avatars presented in the context of satiety sweat as compared to fasting sweat (by trend within the P3b) or cotton (P3a: Chemosensory Stimulus: F(2, 38) = 3.75, *p* = 0.041, η^2^_p_ = 0.165, power = 0.650; satiety sweat vs. cotton: t (19) = 3.16, *p* = 0.005, d = 0.706, power = 0.850; satiety sweat vs. fasting sweat: t (19) = 2.41, *p* = 0.027, d = 0.538, power = 0.626; P3b: Chemosensory Stimulus × Electrode Position: F(2, 38) = 4.84, *p* = 0.014, η^2^_p_ = 0.203, power = 0.767; nested effects: Chemosensory Stimulus within Pz: F(2, 38) = 3.65, *p* = 0.035, η^2^_p_ = 0.161, power = 0.638; satiety sweat vs. fasting sweat: t (19) = 1.87, *p* = 0.077, d = 0.418, power = 0.427; satiety sweat vs. cotton: t (19) = 2.95, *p* = 0.008, d = 0.660, power = 0.799, Figure 6B).

##### Latencies

While the N1 components’ latencies were unaffected by the avatars’ body shape and the type of the chemosensory context stimulus (all *p_s_* ≥ 0.483), the latencies of the P2 component were affected in a similar way as the N1 amplitudes (see above). The P2 component appeared with the shortest latency, whenever the combination of the social stimuli was inconsistent. When participants were presented with underweight avatars in the context of satiety sweat, they displayed shorter P2 latencies as compared to being presented with overweight avatars in the same context (Body Shape × Chemosensory Stimulus: F(2, 38) = 3.79, *p* = 0.032, η^2^_p_ = 0.166, power = 0.655; Body Shape within satiety sweat: F(1, 19) = 9.00, *p* = 0.007, η^2^_p_ = 0.321, power = 0.811). Consistently, participants showed shorter P2 latencies in response to overweight avatars presented either in the context of cotton or in the context of fasting sweat compared to overweight avatars in the context of satiety sweat (based on the same interaction Body Shape × Chemosensory Stimulus; Chemosensory Stimulus within overweight avatars: F(2, 38) = 5.24, *p* = 0.010, η^2^_p_ = 0.216, power = 0.802; satiety sweat vs. fasting sweat: t (19) = 2.06, *p* = 0.053, d = 0.461, power = 0.499; satiety sweat vs. cotton: t (19) = 3.44, *p* = 0.003, d = 0.769, power = 0.903).

Participants further showed a generally shorter P3a latency in response to avatars presented in the context of fasting sweat as compared to satiety sweat and cotton (Chemosensory Stimulus: F(2, 38) = 3.99, *p* = 0.031, η^2^_p_ = 0.174, power = 0.680; satiety sweat vs. fasting sweat: t (19) = 2.60, *p* = 0.018, d = 0.581, power = 0.693; cotton vs. fasting sweat: t (19) = 2.78, *p* = 0.012, d = 0.621, power = 0.750.)

The P3b component’s latency was unaffected by the avatars’ body shape and the type of the chemosensory context stimulus (all *p_s_* ≥ 0.242). For a comprehensive overview of the ANOVA results see Table 4.

##### Low-Resolution Electromagnetic Tomography Analysis

LORETA revealed identical areas of maximum cortical activation in response to overweight and underweight avatars (across all chemosensory conditions) throughout all processing stages (N1, P2, P3a, and P3b). During the earliest stimulus processing stage (N1 time frame), the most pronounced activation was evident within the right primary visual cortex (BA17), while during all other processing stages (P2, P3a, and P3b) the strongest activity was found within the right angular gyrus (BA39).

## 4. Discussion

The current study is the first to examine neural responses to human chemosensory cues related to fasting and satiety. In comparison with chemosensory fasting cues, satiety cues are processed with enhanced neuronal activity throughout all processing stages, indicating augmented attention and pronounced stimulus significance. It is important to note that the here-presented study investigated the effects of overnight short-term fasting instead of long-term fasting or starvation. Each of these might have different effects on body odor quality. While overnight short-term fasting seems not to reveal important information about the senders’ nutritional state, prolonged daily fasting as well as therapeutic fasting for several days might increase the attractiveness of body odor [16], whereas chronic starvation might be related to a decreased attractiveness of body odor [14,15]. Moreover, in the present study, it could be shown that the processing of any body shape presented in the context of satiety cues resembles that of overweight body shapes, suggesting that satiety-related cues indeed communicate a well-fed nutritional status. In line with our hypotheses, the results show that human sweat-derived volatiles convey information about the nutritional status, which is perceived and processed by the receiving individual, and that this information shapes the processing of related social stimuli of another modality.

The presentation of the sweat samples was not accompanied by the perception of a distinct odor, as most participants could not differentiate cotton pads with and without sweat. Accordingly, the olfactory evaluations of fasting and satiety sweat did not differ. Furthermore, the participants did not judge the BMI of fastened and satiated donors’ sweat any differently, suggesting that the samples’ smell did not activate concepts related to fasting and satiety. Several studies have repeatedly reported human chemosensory stimuli as being difficult to detect or recognize, although they are processed as relevant information in the human brain [39,40,41]. This phenomenon is considered to reflect the activation of neuronal priming processes, easing behavioral and physiological adaptations in response to significant social information transmission.

Regarding the CSERPs, during early (P1, P2), and late processing stages (P3), participants responded with enlarged amplitudes to sweat obtained from satiated individuals compared to sweat of the same individuals obtained after having undergone short-term (12 h) fasting. The early CSERP components are generally thought to reflect early stimulus encoding and are affected by stimulus features such as stimulus complexity and the allocation of attention [42,43]. It can thus be assumed that the increased neuronal activity accompanying the encoding of chemosensory satiety cues is either due to the increased information content of chemosensory satiety as compared to fasting cues, thereby reflecting metabolic activity, or to an activation of attentional resources in response to chemosensory cues of satiety.

The intensified strength of the early processing of satiety sweat is accompanied by activation within BA6 (right middle frontal gyrus, P1 time frame) and BA39 (angular gyrus P2 time frame). A recent meta-analysis confirmed that the right middle frontal gyrus is crucially activated during the processing of (emotionally) significant human chemosignals [44]. An activation of the angular gyrus (BA39) in response to human chemosignals is discussed as being related to shifting attention towards behaviorally relevant signals [45,46], and to the detection of another individual’s presence [47].

Regarding late evaluative stimulus processing, a pronounced P3 amplitude, as evident in response to satiety sweat within the current study, has been related to the special significance of a given human social chemosignal: Such amplified evaluative processing has been shown during exposure to anxiety as well as aggression-related sweat samples [39,48,49]. The most pronounced neural activation within the P3 time frame occurred at BA19 of the inferior temporal gyrus, close to the fusiform gyrus (BA 19/37). Activation of the fusiform gyrus during the processing of human chemosignals has repeatedly been shown (e.g., [40,50,51]), indicating the recognition of the “human quality” transmitted by these signals.

Taken together, the pattern of early and late processing suggests that satiety sweat contains rich and complex social information about other individuals which is readily attended to and processed as important social information in the human brain. In accordance with the present results, chemosensorily transmitted health-related information ranges from information about nutritional status [16] to information about the current immune status [52]. The current results show that the human brain processes such information even below the threshold of conscious stimulus detection. It is concluded that brain processing changes due to chemical communication are not necessarily translated into measurable changes in conscious perception and evaluation. However, according to the CSERP and LORETA analyses, chemosensory satiety cues are considered to be processed as a significant type of social information, increasing alertness and prompting perceptual and response systems for an optimum preparedness for social adjustment.

The analyses of the visual ERP in response to avatars of varying body-size revealed enhanced processing of overweight avatars throughout all processing stages (N1, P2, P3a, P3b amplitudes) on a descriptive level, and especially during early processing (P2 amplitude, with an effect approaching significance). This pattern emerged irrespective of the chemosensory context. Similar effects have repeatedly been shown in response to visual stimuli depicting obese as compared to underweight individuals [33,53,54,55]. Intriguingly, the context of satiety sweat appears to shift the brain towards processing any given body shape as “overweight”, since both early (P2) and late processing (P3a, P3b) ERP amplitudes upon presentation of avatars were larger in the context of satiety compared to fasting sweat, irrespective of the avatar’s actual body shape. This process is not reflected in an altered conscious evaluation of body shapes; instead, human satiety chemosignals seem to facilitate both more stimulus-driven attentional (P2, P3a) as well as evaluative processing (P3b) of other individuals’ bodies. Thus, pre-attentive as well as attentional resources are activated, facilitating but not determining perceptual and response selection strategies. The phenomenon of chemosignals overriding the social information from the visual modality has already been demonstrated with chemosignals derived from emotion-related sweat, indicating a processing advantage for social information from the chemosensory over other modalities [22,56].

The N1 in response to the avatars is affected in a different manner by the chemosensory context: Its amplitude is generally most pronounced whenever the participants are exposed to any incongruent combination of social stimuli, that is, pictures of overweight avatars in the context of fasting sweat, or pictures of underweight avatars in the context of satiety sweat. This pattern indicates the processing of incongruent information and expectancy violation: Because the chemosensory context is present prior to picture presentation, it might subconsciously prime the visual exposure to an individual with either a congruently well-nourished (overweight) or fasted (underweight) body shape. The processing of incongruent cross-modal social perception [57,58] as well as expectancy violation (e.g., [59]) has repeatedly been shown to result in an enhanced attention allocation as reflected in an enlarged N1 amplitude. The results of shorter latencies of the visual P2 in response to incongruent stimulus combinations, as evident within the current study, is in line with this notion, suggesting that not only the strength but also the speed of early processing might be affected.

We here addressed the recipient’s chemosensory information obtained via sweat samples obtained from donors in their fasted vs. satiated state; it is also of interest in further studies to account for an interesting role of metabolic information conveyed to our olfactory system. Interestingly, primary olfactory neurons, the olfactory mucosa, and also the olfactory bulb contain receptors [60] for neuropeptides with catabolic (anorexigenic) actions in the brain such as insulin and leptin, for those conveying anabolic (orexigenic) information such as neuropeptide Y and orexin, and also nutrients (glucose, amino acids).

A limitation of the here presented study is the relatively small sample size, not allowing for further investigation of modulating effects due to certain receiver characteristics. For example, further studies could separate odor detectors from non-detectors; thereby investigating whether conscious stimulus detection changes the neuronal differentiation of chemosensory satiety and fasting cues. In addition, possible gender differences in stimulus processing could be investigated (for a discussion of gender differences in chemosensory communication see [49,61]).

## 5. Conclusions

Taken together, the current study shows for the first time that humans communicate subtle changes of the metabolic state and that short-term fasting for 12 h can be differentiated from the consumption of a standard breakfast. Second, it could be shown that the processing of chemosensory information regarding a just-consumed meal requires more neuronal resources than the processing of chemosensory information of overnight fasting. In line with the animal literature [10,11,12,13,14,15], it is here proposed that information about the metabolic state contains important cues on the health status and might help the perceiver to select social response strategies related to behavioral approach or withdrawal. Finally, in comparison with visual social information, chemosensory social information seems to be of high importance for the integration of conflicting sensory information to a coherent perceptual concept.

## Figures and Tables

**Table 1:** Weight and caloric content of the standardized breakfast’s components.

Components	Weight	Caloric Content
White bread	70 g	173 kcal
Honey	20 g	60 kcal
Jam	25 g	56 kcal
Cream cheese	33 g	85 kcal
Chocolate bar	21 g	92 kcal
Orange juice	0.33 L	142 kcal

**Table 2:** Descriptive difference values of the sweat donors’ BMI estimation and the odor ratings.

	Estimated Donors’ BMI (kg/m^2^)		Ratings							
			Intensity		Pleasantness		Unpleasantness		Familiarity	
	M	SD	M	SD	M	SD	M	SD	M	SD
Delta SC	−13.0	27.9	1.00	1.53	−0.53	2.17	0.68	2.14	0.37	2.09
Delta FC	−16.9	23.8	0.84	2.48	−0.47	1.78	0.84	2.63	0.89	2.81

Notes. Delta SC = difference satiety minus cotton, Delta FC = difference fasting minus cotton.

**Table 3:** Analyses of variance of the visual event-related potentials’ amplitudes: Summary of main effects, interactions, and follow-up pairwise comparisons.

Effect	N1 (Fz, Cz)	P2 (Cz, Pz)	P3a (Cz, Pz)	P3b (Cz, Pz)
BS		OA > UA (*)		
CS		sat > cot *	sat > cot **	
			sat > fas *	
EP			Pz > Cz *	Pz > Cz ***
BS × CS	UA > OA in sat (*)			
	OA > UA in fas (*)			

	cot > sat in OA (*)			
	fas > sat in OA *			
CS × EP				sat > cot in Pz **
				sat > fas in Pz (*)
BS × CS × EP	OA > UA in Fz in fas **			
	OA > UA in Cz in cot (*)			
	UA > OA in Cz in sat *			

	cot > sat in Cz in OA *			
	fas > sat in Cz in OA *			

Notes. BS = Body Shape, CS = Chemosensory Stimulus, EP = Electrode Position, UA = underweight avatars, OA = overweight avatars, fas = fasting sweat, sat = satiety sweat, cot = cotton, *** *p* < 0.001, ** *p* < 0.01, * *p* < 0.05, (*) *p* < 0.10.

**Table 4:** Analyses of variance of the visual event-related potentials’ latencies: Summary of main effects, interactions, and follow-up pairwise comparisons.

Effect	N1 (Fz, Cz)	P2 (Cz, Pz)	P3a (Cz, Pz)	P3b (Cz, Pz)
CS			fas < cot *	
			fas < sat *	
EP	Fz < Cz (*)	Cz < Pz *		Cz < Pz *
BS × CS		UA < OA in sat **		
		cot < sat in OA **		
		fas < sat in OA (*)		

Notes. BS = body shape, CS = chemosensory stimulus, EP = electrode position, UA = underweight avatars, OA = overweight avatars, fas = fasting sweat, sat = satiety sweat, cot = cotton, ** *p* < 0.01, * *p* < 0.05, (*) *p* < 0.10.

## Data Availability

The datasets generated for this study are available upon request from the corresponding author.

## Appendix A

**Table A1:** Results of a 2 × 3 × 3 ANOVA depicting the topographical dominance of the visual ERP components.

	ANOVA (Electrode Position)	*t*-Tests		M [µV]	SD [µV]
N1	F(2, 38) = 9.58, *p* = 0.001, η^2^_p_ = 0.335, power = 0.972	Fz vs. Pz t (19) = 3.44, *p* = 0.003, d = 0.769, power = 0.903 Cz vs. Pz t (19) = 3.62, *p* = 0.002, d = 0.809, power = 0.929	Fz: Cz: Pz:	−4.91 −4.70 −3.32	1.77 2.53 2.01
P2	F(2, 38) = 8.71, *p* = 0.003, η^2^_p_ = 0.314, power = 0.957	Fz vs. Cz t (19) = 6.33, *p* < 0.001, d = 1.415, power > 0.999 Fz vs. Pz t (19) = 3.02, *p* = 0.007, d = 0.675, power = 0.817	Fz: Cz: Pz:	5.62 8.01 7.96	4.62 5.56 4.59
P3a	F(2, 38) = 23.78, *p* < 0.001, η^2^_p_ = 0.556, power > 0.999	Fz vs. Cz t (19) = 6.15, *p* < 0.001, d = 1.374, power > 0.999 Fz vs. Pz t (19) = 5.80, *p* < 0.001, d = 1.297, power > 0.999	Fz: Cz: Pz:	4.96 7.46 9.14	4.44 5.51 4.95
P3b	F(2, 38) = 43.31, *p* < 0.001, η^2^_p_ = 0.695, power > 0.999	Fz vs. Cz t (19) = 5.08, *p* < 0.001, d = 1.136, power = 0.998 Fz vs. Pz t (19) = 7.68, *p* < 0.001, d = 1.716, power > 0.999 Cz vs. Pz t (19) = 5.58, *p* < 0.001, d = 1.247, power > 0.999	Fz: Cz: Pz:	5.37 8.04 10.82	4.11 5.11 4.72
